# Pedagogical Approaches and Learning Activities, Content, and Resources Used in the Design of Massive Open Online Courses (MOOCs) in the Health Sciences: Protocol for a Scoping Review

**DOI:** 10.2196/35878

**Published:** 2022-05-30

**Authors:** Øystein Røynesdal, Jeanette H Magnus, Anne Moen

**Affiliations:** 1 Department of Teacher Education NLA University College Bergen Norway; 2 Institute for Health and Society Faculty of Medicine University of Oslo Oslo Norway; 3 Center for Global Health Faculty of Medicine University of Oslo Oslo Norway; 4 Institute for Health and Society University of Oslo Oslo Norway

**Keywords:** MOOC, scoping review, collaborative learning, PhD, postgraduate, education, health sciences, massive open online course

## Abstract

**Background:**

Developing online, widely accessible educational courses, such as Massive Open Online Courses (MOOCs), offer novel opportunities to advancing academic research and the educational system in resource-constrained countries. Despite much literature on the use of design-related features and principles of different pedagogical approaches when developing MOOCs, there are reports of inconsistency between the pedagogical approach and the learning activities, content, or resources in MOOCs.

**Objective:**

We present a protocol for a scoping review aiming to systematically identify and synthesize literature on the pedagogical approaches used, and the learning activities, content, and resources used to facilitate social interaction and collaboration among postgraduate learners in MOOCs across the health sciences.

**Methods:**

We will follow a 6-step procedure for scoping reviews to conduct a search of published and gray literature in the following databases: Medline via Ovid, ERIC, SCOPUS, Web of Science, and PsychINFO. Two reviewers will screen titles, abstracts, and relevant full texts independently to determine eligibility for inclusion. The team will extract data using a predefined charting form and synthesize results in accordance with the Preferred Reporting Items for Systematic reviews and Meta-Analyses extension for Scoping Reviews checklist.

**Results:**

The scoping review is currently ongoing. As of March 2022, we have performed initial data searches and screened titles and abstracts of the studies we found but revised the search string owing to inaccurate results. We aim to start analyzing the data in June 2022 and expect to complete the scoping review by February 2023.

**Conclusions:**

With the results of this review, we hope to report on the use of pedagogical approaches and what learning activities, content, and resources foster social and collaborative learning processes, and to further elucidate how practitioners and academics can harvest our findings to bridge the gap between pedagogics and learning activities in the instructional design of MOOCs for postgraduate students in the health sciences.

**International Registered Report Identifier (IRRID):**

DERR1-10.2196/35878

## Introduction

Developing online, widely accessible educational courses, such as Massive Open Online Courses (MOOCs), offer novel opportunities to advance academic research and the educational system in resource-constrained countries [1,2]. A PhD proposal or research protocol is one of the first important academic texts aspiring researchers write worldwide [3], however; there is little emphasis on developing academic writing at the postgraduate level [4,5]. To facilitate social equity in the health sciences, an accessible course focusing on PhD proposal writing could offer opportunities for students from low- and middle-income countries (LMICs) to increase their writing capacity and successfully compete for PhD scholarships and scholarly positions at research-intensive higher education institutions (HEIs) [2,6].

MOOCs are “*online* courses designed for large numbers of participants that can be accessed by anyone anywhere as long as they have an internet connection (*massive*), are *open* to everyone without entry qualifications, and offer a full/complete course experience online for *free*” [7]. As such, a MOOC brings together participants from diverse backgrounds and disciplines worldwide with a common interest to learn and coconstruct knowledge in a socially networked, nonformal, computer-mediated learning environment [8,9].

Traditionally, there are 2 main types of MOOCs depending on the pedagogical approach underpinning the design, affordances of the course platform, and the degree of openness (nonprofit or for profit) [10,11]. A cMOOC has a social learning, or connectivist approach [12]. In cMOOCs, active social interaction and collaboration is viewed as required to coconstruct knowledge as a member of an active open, online community [11,13]. Through the learning activities, “learners decide their own objectives, share their knowledge and collaboratively build their ideas and artifacts” [10]. An xMOOC uses an individual learning or cognitive behavioral approach with didactic or transmission models of teaching, with somewhat limited openness (often for profit) and less emphasis on learners’ coconstruction of knowledge [10,14]. Here, the teaching methods often include information transmission, computer-marked assignments, and peer assessment [10]. Other more recent MOOCs use a blended-learning approach, also referred to as hybrid MOOCs (eg, bMOOCs and smMOOCs) combining elements from the different pedagogical approaches [11,15].

Although literature reviews have summarized the use of design-related features and principles of different pedagogical approaches [8,10,15-18], the quality of the learning experiences in MOOCs have been questioned [13,19]. Common learning activities of most types of MOOCs include video or face-to-face lectures, blogs, discussion forums, social networks, lecture notes, PowerPoint slides, and PDFs [10]. However, in cMOOCs or blended-learning MOOCs, where social interaction and collaboration is anticipated to foster key learning throughout or in parts of the course, studies have demonstrated that there are limited learning activities in which social learning *could* take place [13,19]. In addition, a review of cMOOCs and xMOOCs found that only one-third of them had material or resources, which learners viewed relevant for the learning outcome [19]. Considering the dynamic and complex individual learning process in a computer-mediated setting [9], it is important that learning activities, content, and resources reflect an a priori pedagogical approach underpinning the MOOC design, and that the materials offered are viewed by learners as helpful and relevant to achieve the stated learning objectives [13].

In this paper, we present a protocol for a scoping review aiming to systematically identify and synthesize literature on the pedagogical approaches used, and the learning activities, content, and resources used to facilitate social interaction and collaboration among postgraduate learners in MOOCs across the health sciences.

## Methods

### Methods Overview

We will follow the 6-step systematic procedure for scoping reviews as outlined by Arksey and O'Malley [20]. This procedure includes (a) identifying the research question; (b) identifying relevant studies; (c) selecting studies; (d) charting the data; (e) collating, summarizing, and reporting the data; and (f) an optional consultation exercise. We will also use the PRISMA-ScR (Preferred Reporting Items for Systematic reviews and Meta-Analyses extension for Scoping Reviews) checklist to further guide what we report in this protocol and the final review [21] while adhering to recommendations made in recent methodological scoping review papers that advance the procedure proposed by Arksey and O’Malley [22-24].

As the findings of the proposed scoping review can inform a potential revision of the MOOC *How to Write a PhD Proposal*, we account for the theoretical positioning underpinning the work to develop this MOOC and the pedagogical approach used in the design phase. The work aligns with the knowledge creation metaphor of learning*,* where learning is viewed “as a process of knowledge creation which concentrates on mediated processes where common objects of activity are developed collaboratively” [25]. In a computer-mediated online learning setting, such as an MOOC, an underlying principle of the knowledge creation approach to learning is that each learner’s effort to advance knowledge and skills occurs in, and benefits from, participation in the social online community [25,26]. This understanding is in line with the pedagogical approach of cMOOCs and the blended learning approaches used in MOOCs. However, when embarking on developing the MOOC in PhD proposal writing, we had to adjust the thinking underlying the course design to accommodate the blended learning approach integrated in the online learning platform FutureLearn. Although we designed the key activities in accordance with the social knowledge creation approach to learning, the blended learning principles integrated in the FutureLearn platform also accommodated other ways of learning (eg, acquisition learning [25]).

Each learner who registers for the MOOC on PhD proposal writing is assumed to have a research idea, but to develop the research idea into a plan or proposal (the common object of activity), the learners are encouraged to actively participate in the key learning activities or tasks that require social interactions and extensive collaboration with other learners (eg, reviewing the work of others together with other participants). Through these forms of feedback and collaborative work, participants could learn from others by reading the work of others and integrating key elements of PhD proposal writing into their own work.

To ensure consistency throughout this scoping review, the lead researcher will spearhead the work during each stage of the review process [27]. We will set up an iterative process to ensure a well-crafted scoping review that demonstrates procedural transparency and methodological rigor [21,22]. We are a research team with experience in conducting exploratory research and various types of literature reviews in the fields of medicine, nursing, education, and sports science. Throughout the process, we will also track refinements of the proposed protocol [22].

### Identifying the Research Question

The first step of the systematic scoping review process involves identifying and defining the parameters of the research question [20]. This is usually done a priori to guide the literature searches, followed by an iterative process of refinement based on familiarization with the identified research evidence [27]. Our preliminary research question is “What are the pedagogical approaches, and the learning activities, content, and resources used to foster social interaction and collaborative learning processes when designing MOOCs for postgraduate students in the Health Sciences?” We designed our research question to be broad, to generate a large but also manageable pool of data to establish an overview of the topic investigated [20,22]. We have formulated the following subquestions to further guide our scoping inquiry:

1. What are the pedagogical approaches used to guide the design of MOOCs in the health sciences?
2. What are the learning activities, content and resources used in MOOCs to facilitate social interaction and collaboration?
3. What are the social, collaborative learning activities, content, and resources do participants report as helpful and relevant in MOOCs?

Defining and operationalizing the concepts of a review question is useful to clarify the scope [24]. It guides the subsequent phases of exploration and clarifies the final review report [28]. For this study protocol, the term “design” refers to the development phase of MOOCs preceding the online launch. We define postgraduate studies as academic studies above the master’s level (eg, MA, MSc, and MPhil) or equivalent (eg, MD). As such, we refer to postgraduate students as a group of people who have completed their master’s thesis or similar academic degrees. When using the phrase “PhD proposal,” we refer to a research protocol’s specific format, genre, and style of writing needed when developing a PhD project proposal or presenting a stringent research plan, and, as such, preparing for work that meets the requirements for writing research articles or manuscripts of project results.

### Identifying Relevant Studies

#### Overview

The second step of the scoping review is to identify relevant literature [20]. Clear definitions and operationalization of the research question, and a specification of the inclusion criteria that underpins the search strategy are necessary [29]. However, developing the search strategy is an iterative and reflexive process that requires assessment and refinement based on both the pilot searches and the materials yielded from the searches [27]. We will start with a comprehensive and broad search including published and gray literature. In line with the search guidelines developed by Aromataris and Riitano [29], we will (i) explore published literature in electronic databases, (ii) hand-search reference lists in the identified literature, and (iii) review the table of contents in a few key journals. We will search gray literature (i) by hand-searching selected organization, government, and conference websites, (ii) using selected gray literature databases, and (iii) using online search engines.

To ensure breadth and optimize consistency of the literature searches, an experienced research librarian will build, structure, and conduct all literature searches across databases [29]. As the search may yield a wide variety of studies across both published and gray literature, we will not exclude any published literature on the basis of the study design.

#### Search Strategy

We will adapt a Population (or participants)/Concept/Context (PCC) mnemonic and use the Boolean logic of “AND” and “OR” to build the various searches across databases. Findings in each database and the full combination of key terms used will be registered for each individual grid. We will pilot the search strategy using a 3-step approach, which includes (a) an initial search of a key database and analyze alternative terms and index or MeSH terms used to describe the concepts and (b) search the chosen database for the keywords and index terms identified [29]. Based on the piloting of our search strategy and familiarization with the research field, we may refine or add keywords and index or MeSH terms before searching the remaining databases. We will report the full details of our searches in the electronic databases as an appendix in the main scoping review paper [21].

Our search will cover the period from 2004 to March 2022. Searching for literature before 2004 is not likely to yield relevant results since the first MOOC was launched in 2008. Written material to be included in the scoping review is, for practical and financial reasons, limited to English and the Nordic languages. Using the reference management software EndNote, we will collate and organize all studies extracted per database [30] and use the integrated algorithm in EndNote to screen for duplicate references [31]. One reviewer will then carry out an initial screening of all references identified in the literature search to exclude clearly irrelevant studies; for example studies or reports emphasizing online courses targeting children, primary school students, or high school students. If the reviewer has any doubt about whether to exclude a study or report, it will be included for further scrutiny by other reviewers at a later stage.

#### Electronic Databases

We will search the multidisciplinary electronic databases (eg, SCOPUS) illustrated in Table 1. Some electronic databases have a somewhat similar interface but index subject headings differently. The research librarian will tailor our search of each database to include the specific subject headings listed. 

**Table 1:** A preliminary overview of electronic databases for the published literature search.

Name	Scope
Education Resources Information Centre	International literature database within pedagogy
Medline via Ovid	Medicine, nursing, dentistry, biomedical research
Web of Science	Cross-disciplinary, international database with mainly articles
SCOPUS	Large bibliographic database containing abstracts and citations for academic journal articles (peer-reviewed journals in medical, technical, and social sciences, including the arts and humanities)
PsycINFO via OVID	Centered on psychology and the behavioral and social sciences

#### Hand-Searching Reference Lists and Key Journals

We will investigate the bibliographies of literature findings and the table of contents in a few key journals that publish studies on pedagogical practices and instructional designs in MOOCs for additional relevant papers. A special emphasis is on ensuring that we also select journals that are not well indexed in available commercial or public databases to avoid missing out on potentially eligible studies [32].

#### Gray Literature

Searching for gray literature in repositories that specifically target this form of literature is useful to ensure breadth and limit the publication bias in literature reviews [32]. We define gray literature as conference proceedings and literature produced at all levels of government, academic, business, and industry, available in electronic and printed formats not controlled by commercial publishing houses. This includes any paper or study not formally published or peer reviewed; that is, reports, working papers, theses and dissertations, conference posters and presentations, unpublished protocols and guidelines, market reports, government documents, and white papers [33].

We will search a selection of the gray literature databases illustrated in Table 2. As these repositories may not have advanced search modes, we will use a few carefully selected critical key terms from our search grid [29]. To ensure comprehensiveness, we will also search the unpublished literature on the internet using online search engines, such as Google, in their incognito, advanced search mode with a stringent search query based on the terms used in the search string. Using relevance filtering, we will further limit the assessment to the first 50 hits per combination of key terms [34]. We will list the potential findings using EndNote [27].

Synthesizing gray literature and gray data in a systematic way is a difficult task [35]. To ensure replicability, we will list the findings from each stringent combination of key terms used in each database and add as an appendix to the main review paper.

**Table 2:** A preliminary overview of electronic gray literature databases for the literature search.

Name	Scope
Open-Grey	An open access gray literature database that includes technical or research reports, doctoral dissertations, some conference papers, some official publications, and other types of gray literature. OpenGrey covers Science, Technology, Biomedical Science, Economics, Social Science, and Humanities
ProQuest Dissertations and Theses	ProQuest has one of the largest collections of electronic theses and dissertations available worldwide
Google Scholar	International database that indexes the full text or metadata of scholarly literature across an array of publishing formats and disciplines, including educational science

### Study Selection

The third step of the scoping review is an iterative assessment of the identified literature using the defined eligibility criteria [20,21]. A flowchart will illustrate the selection process of the literature [21]. It will specify the number of duplicates that are removed and the references excluded on the basis of either the title or abstract or the full text [34].

We will include all studies that report on conceptual or theoretical frameworks or pedagogical approaches used to guide design considerations; selection or structuring of content, learning activities, and resources that are assumed to stimulate social interaction and collaboration; or participant views of learning material anticipated to foster social learning in cMOOCs and hybrid MOOCs. We will exclude studies or reports that describe learning material, content, or resources in xMOOCs or comparisons of online learning platforms, grading, or assessment of learner achievements, or outcome or operational or technical issues (eg, weekly supervision of courses and considerations during the launch of MOOCs), as well as studies that target participants at the undergraduate level at HEIs.

We will review studies using an iterative 2-step approach [24]. The first step involves importing all references into the app-based literature review program Covidence to screen the title and abstract in accordance with inclusion or exclusion criteria. To do so, we will hold team meetings at the beginning, midpoint, and end to discuss progress as well as any challenges and uncertainties (the number of references identified dictates the number of meetings required) related to study inclusion. If necessary, the search strategy will be refined. The second step involves the full reading of the retained studies and reports to decide on final inclusion. We will first extract the references from Covidence back into EndNote to acquire the full-text version of the studies or reports before the references are imported back to Covidence for the full read.

During the study selection process, two reviewers will screen titles, abstracts, and relevant texts independently to determine eligibility for inclusion in this scoping review [21,24]. If the first reviewer chooses to include a reference, the second reviewer will verify his or her selection of the specific study. If there are divergences between reviewers, a third reviewer will be consulted.

We will use the Mixed Methods Appraisal Tool (MMAT) to appraise the quality of the published literature identified [36]. The MMAT tool includes an extensive assessment procedure of qualitative, quantitative, and mixed methods studies. To appraise the quality of gray literature, we will use the AACODS (Authority, Accuracy, Coverage, Objectivity, Date and Significance) checklist [37]. We will not perform a full assessment of methodological robustness as we argue that it is more important and appropriate for a scoping review to capture breadth in the literature rather than a limited sample of methodological quality-assessed studies used for systematic reviews.

### Data Charting

The fourth step of the scoping review process entails organizing and synthesizing the data in accordance with key themes [20]. Based on initial discussions, the research team plans to extract and chart a mixture of general information about the studies or reports in this scoping review. More specifically, we will extract the names of the authors, year of publication, study location, type of document source, aims, and any assessments of target groups or participants. Underpinned by the knowledge creation approach to learning, we will extract specific information from each study and report related to conceptual or theoretical frameworks and pedagogical approaches; selection or structuring of social, collaborative learning activities, content, and resources; and participant evaluations of any material viewed as helpful in cMOOCs or hybrid MOOCs to stimulate interaction and collaboration.

We will develop a predefined Microsoft Excel data charting form. Refinement of the charting form may be necessary, as data extraction is an iterative process involving continual assessment, evaluation, and updating [34]. To ensure consistent data extraction, the lead researcher will undertake a pilot of the form on a random sample of 5-10 studies. In consultation with the full research team, we will modify, if necessary, to mitigate inconsistencies and uncertainties of use to ensure coherence with the research question [24,27].

### Collating, Summarizing, and Reporting the Results

The fifth phase of the scoping review process entails summarizing and reporting findings in a clear and consistent way [20]. Throughout the reporting phase, we will follow the recommendations of the PRISMA-ScR guidelines when writing up the final review [21]. We will summarize and describe each identified study’s characteristics (eg, author name, study location, and year of publication), as well as the pedagogical and theoretical or conceptual approach used in the design of the MOOC, the learning activities, content, and resources adopted and any participant evaluation of materials used in MOOCs. We will then report the output with an emphasis on describing how the findings relate to the subquestions guiding the scoping review before discussing the implications for pedagogical practice and instructional design of MOOCs targeting prospective postgraduate students in the health sciences.

### Consulting Relevant Stakeholders

The sixth stage of the scoping review process is an optional consultation stage [20]. We agree with other researchers who argue that consultation could be useful for knowledge translation [23,24]. We will consider inviting relevant stakeholders, such as international PhD students from LMICs and experts in online, computer-mediated pedagogies, to advice on how to provide appropriate, useable learning resources, and how learning activities or material identified can be used to underpin inclusive, collaborative learning processes among students with diverse backgrounds in a potential revision of the MOOC *How To Write a PhD Proposal.*

To disseminate findings, we seek to publish the scoping review in an international peer-reviewed journal with a scope that includes the use of collaborative pedagogies in computer-mediated and online learning environments. We also aim to disseminate the findings to professionals and other colleagues in postgraduate studies to potentially inspire improved instructional designs of MOOCs.

## Results

The scoping review is currently ongoing. As of March 2022, we have completed an initial data search and screened the title and abstract of all references. However, as the initial search captured a large bout of irrelevant published studies beyond the field of study, we decided to revise the search string across all databases in a second literature search. We aim to start analyzing the data in June 2022 and expect to complete the scoping review by February 2023. The proposed scoping review will inform a potential revision of the MOOC *How To Write A PhD Proposal.* The MOOC is currently delivered on the FutureLearn platform by colleagues from the University of Oslo. The MOOC is funded by the University in Oslo and the NORPART: EXCEL SMART program (2016/10213).

## Discussion

We have developed a scoping review protocol aimed at investigating the pedagogical approaches used, and the type of learning activities, content, and resources used to foster social interaction and collaboration when designing MOOCs at postgraduate level in the health sciences.

The findings of the proposed scoping review may have direct implications for academic staff and professionals working with the instructional design or pedagogical practices of MOOCs or in other online, collaborative courses. First, it could directly influence the way key learning activities and tasks are structured or designed in cMOOCs or blended-learning MOOCs. As the proposed scoping review can inform a potential revision of the MOOC in PhD proposal writing, where the first runs had a wide range of participants from both high-income countries and LMICs worldwide, identifying how social or collaborative learning activities and resources can be adjusted to accommodate the needs of students from LMICs or resource-constrained educational system (eg, internet connectivity issues) can result in a higher completion rate of students enlisting for the course [38]. To this end, it could influence the extent of which participants improve the quality of their PhD proposal writing and thus have social implications for the recruitment of candidates to PhD programs in increasingly internationalized research education programs across the globe. Second, understanding how current learning activities, content, or resources can be modified to underpin inclusive, collaborative learning processes among participants with diverse backgrounds, we could potentially improve course designs and the quality of the individual learning experience in MOOCs using a social learning or blended learning approach [19].

We followed the PRISMA-ScR to inform what we reported throughout this protocol [21]. Very few scoping reviews are reported, and even fewer published [39]. We contend that publishing this protocol can contribute to increased transparency and methodological rigor of the final review and future review studies, as many scoping reviews often lack sufficiently detailed descriptions of search strategies and procedures [30,39]. In accordance with the PRISMA-ScR [21], we have therefore detailed our research strategy, the theoretical position, and how this could influence the specific phases of the review procedure. We have also described our search strategy for both published and gray literature databases in a detailed way. As there is usually no methodological assessment of included studies in scoping reviews, using quality appraisal tools such as the MMAT [36] and the AACODS checklist [37] can further increase the rigor, accuracy of interpretation of data, and synthesis of findings [21,23,27]. Through these measures, the potential for reproducibility increases and allows readers of the proposed scoping review to *fully* engage with and assess the assumptions underpinning the work, as well as the specific steps of the scoping review procedure [21].

However, there are limitations related to the scoping review procedure as described in this protocol. As the research team comprises relatively few members, there is a risk that the literature searches can produce an excessive amount of data, which is unfeasible to process and analyze. To mitigate this, we have recruited a research librarian to compile accurate but comprehensive search strategies. If the body of literature proves unmanageable or the database searches are inaccurate, we will further adjust the search string and continuously assess the scope of our search.

## Abbreviations

HEI: higher Education Institutes
LMIC: low- and middle-income country
MMAT: Mixed Methods Appraisal Tool
MOOC: Massive Open Online Course
PRISMA-ScR: Preferred Reporting Items for Systematic reviews and Meta-Analyses extension for Scoping Reviews
